# T1 Relaxation Times in the Cortex and Thalamus Are Associated With Working Memory and Information Processing Speed in Patients With Multiple Sclerosis

**DOI:** 10.3389/fneur.2021.789812

**Published:** 2021-12-03

**Authors:** Christian Thaler, Isabelle Hartramph, Jan-Patrick Stellmann, Christoph Heesen, Maxim Bester, Jens Fiehler, Susanne Gellißen

**Affiliations:** ^1^Department of Diagnostic and Interventional Neuroradiology, University Medical Center Hamburg-Eppendorf, Hamburg, Germany; ^2^Department of Neurology, University Medical Center Hamburg-Eppendorf, Hamburg, Germany; ^3^Institute for Neuroimmunology and Multiple Sclerosis, University Medical Center Hamburg-Eppendorf, Hamburg, Germany; ^4^APHM La Timone, CEMEREM and Department of Neuroradiology, Marseille, France; ^5^Aix-Marseille University, CNRS, CRMBM, UMR 7339, Marseille, France

**Keywords:** T1 relaxometry, multiple sclerosis, cognitive impairment, quantitative MRI, thalamus, T1 relaxation time

## Abstract

**Background:** Cortical and thalamic pathologies have been associated with cognitive impairment in patients with multiple sclerosis (MS).

**Objective:** We aimed to quantify cortical and thalamic damage in patients with MS using a high-resolution T1 mapping technique and to evaluate the association of these changes with clinical and cognitive impairment.

**Methods:** The study group consisted of 49 patients with mainly relapsing-remitting MS and 17 age-matched healthy controls who received 3T MRIs including a T1 mapping sequence (MP2RAGE). Mean T1 relaxation times (T1-RT) in the cortex and thalami were compared between patients with MS and healthy controls. Additionally, correlation analysis was performed to assess the relationship between MRI parameters and clinical and cognitive disability.

**Results:** Patients with MS had significantly decreased normalized brain, gray matter, and white matter volumes, as well as increased T1-RT in the normal-appearing white matter, compared to healthy controls (*p* < 0.001). Partial correlation analysis with age, sex, and disease duration as covariates revealed correlations for T1-RT in the cortex (*r* = −0.33, *p* < 0.05), and thalami (right thalamus: *r* = −0.37, left thalamus: *r* = −0.50, both *p* < 0.05) with working memory and information processing speed, as measured by the Symbol-Digit Modalities Test.

**Conclusion:** T1-RT in the cortex and thalamus correlate with information processing speed in patients with MS.

## Introduction

Magnetic resonance imaging is an established tool to help diagnose and monitor inflammatory disease progression in multiple sclerosis (MS) (1). With the imaging contrasts acquired in clinical routine the white matter (WM) lesion load, the presence of contrast-enhancing, and new lesions can be determined. However, there is only a poor association between these imaging parameters and clinical disability in MS (2).

Besides inflammatory processes affecting the WM, also gray matter (GM) involvement has been shown to be extensive in MS and is associated with clinical disability (3–5). Especially diffuse gray matter injury in the thalamus, caused by demyelination and secondary degeneration, is recognized to contribute to cognitive impairment in patients with MS (6, 7). However, these histopathological processes are often subtle and difficult to detect with conventional MRI protocols. Therefore, quantitative MRI techniques have been established in imaging protocols that help to detect and allow the quantification of diffuse WM and GM injury (8–10).

A promising technique to assess microstructural damage within the GM is T1 relaxation time (T1-RT) mapping (11). Cortical T1-RT has been reported to be elevated in patients with MS and is associated with clinical disability (12–15). Still, studies focusing on changes of T1-RT in the GM, especially the cortex and the thalamus, using 3-dimensional T1 mapping are limited. With the double inversion-contrast magnetization-prepared rapid gradient echo (MP2RAGE) sequence, introduced by Marques et al., it is now possible to generate T1-RT maps in clinically acceptable scan times with a high in- and through-plane resolution (16).

The aim of this study was to apply the MP2RAGE sequence in patients with MS to investigate T1-RT changes in the cortex and thalamus. We hypothesized that T1-RT alterations in the cortex and thalami correlate with clinical and cognitive impairment.

## Methods and Patients

The study group consisted of 47 patients with relapsing-remitting MS (RRMS) and 17 age-matched healthy controls were enrolled in this monocentric study from February 2014 to November 2014. Inclusion criteria were as follows: age 18–70 years, diagnosis of MS according to the 2010 revised McDonald criteria (1), and absence of neurologic conditions other than MS. Patients with the clinically isolated syndrome (CIS) or primary progressive MS (PPMS) and patients with MRI contraindications were excluded. Inclusion criteria of the age matched-healthy controls were the absence of any thrombotic, vascular, or neurological disease. The healthy control subjects underwent MRI examination solely for research purposes. An overview of the study population is given in Table 1. The study was approved by the local Ethics Committee Hamburg (Ethik-Kommission der Ärztekammer Hamburg) following the guidelines of the Declaration of Helsinki and patients provided written informed consent.

**Table 1:** Demographic, clinical, and conventional MRI data obtained from patients with MS and healthy controls.

	**MS Patients (*n* = 47)**	**Healthy controls (*n* = 17)**	***p***
Age (years)	38.5 ± 10.7	33.3 ± 12.6	0.09
Sex	17 females	9 females	
Disease duration (years)	6.5 ± 6.0	–	
EDSS (*n* = 47)	2 (0–6)	–	
SDMT SD (*n* = 42)	−0.1 ± 1.1	–	
9HPT (sec) (*n* = 42)	18.79 ± 4.26	–	
25FW (sec) (*n* = 40)	4.03 ± 1.14	–	
T2L number	32.3 ± 19.5	–	
T2L volume (ml)	6.80 ± 6.95	–	
NBV (ml)	1466.06 ± 104.46	1550.96 ± 64.34	**0.004**
GMV (ml)	800.13 ± 64.43	843.2 ± 46.23	**0.015**
WMV (ml)	665.94 ± 65.00	707.76 ± 41.34	**0.018**
*T1-RT*			
Cortex (ms)	1360.99 ± 30.76	1352.69 ± 15.51	0.23
Right Thalamus (ms)	1000.02 ± 33.77	1009 ± 28.28	0.39
Left Thalamus (ms)	989.04 ± 32.30	1006.75 ± 24.69	0.05
NAWM/WM (ms)	892.46 ± 35.88	854.76 ± 15.46	**<0.001**

*Mean and SD. The EDSS is displayed as median and range. P-values <0.05 are bold*.

### Clinical Status

Clinical status was assessed for each patient by an MS-specialized neurologist using the Expanded Disability Status Scale (EDSS) according to published guidelines (17). In addition, the oral Symbol-Digit Modalities Test (SDMT), the Nine-Hole Peg Test (9HPT), and the Timed 25-Foot Walk (25FW) were assessed. For the SDMT a SD to age and education matched control cohort was calculated to exclude these confounding factors (18, 19).

### Image Acquisition

All scans were performed on a 3 T MR scanner (Skyra, Siemens Medical Systems, Erlangen, Germany) and the MRI protocol included a sagittal 3-dimensional fluid attenuated inversion recovery (FLAIR) sequence (TE = 390 ms, TR = 4,700 ms, inversion time (TI) = 1,800 ms, 192 slices, FOV = 256 mm, voxel size = 1 mm × 1 mm × 1 mm, flip angle = 120°), a T1w magnetization-prepared rapid gradient echo (MPRAGE) sequence before and after Gadolinium injection (TE = 2.43 ms, TR = 1,900 ms, TI = 900 ms, 192 slices, FOV = 256 mm, voxel size = 1 mm × 1 mm × 1 mm, flip angle = 9°), and a MP2RAGE sequence (TE = 2.98 ms, TR = 5,000 ms, TI 1 = 700 ms, TI2 = 2,500 ms, 176 slices, FOV = 256 mm, voxel size = 1 mm × 1 mm × 1 mm, flip angle = 4°, scan time = 11 min and 17 s). The MP2RAGE sequence was acquired prior to contrast administration.

### Image Analysis

#### Lesion Segmentation

T2 lesions (T2L) were semi-automatically segmented on FLAIR images using the lesion growth algorithm implemented in the toolbox “LST: Lesion Segmentation Tool” (20). For optimal lesion segmentation, the final threshold was set at κ = 0.1. If necessary, lesion outlines were corrected manually by an experienced neuroradiologist with 6 years of experience in MS imaging. FLAIR images and T2L masks were co-registered to the MP2RAGE images using FMRIB's Linear Image Registration Tool [FLIRT; FMRIB Software Library (FSL)] (21).

#### Tissue Segmentation

To avoid misclassification of GM T2L that appeared hypointense on T1w images were filled with intensities from immediately surrounding white matter using FSL's lesion_filling (22). Tissue segmentation was performed on T1w MPRAGE images using the “recon-all” stream from FreeSurfer 5.3 (http://surfer.nmr.mgh.harvard.edu). The T1w MPRAGE images as well as the segmented cortex and thalamus masks were co-registered to the MP2RAGE image using FSL's FLIRT. To avoid misplaced voxels by the co-registration, an additional GM mask was obtained on MP2RAGE images using FSL's FAST and multiplied with the FreeSurfer cortex mask to obtain a consensus mask. Furthermore, a WM mask was obtained from FSL's FAST. In patients with MS, the T2L masks were subtracted from the WM masks to receive a normal-appearing white matter (NAWM) mask in MP2RAGE space.

**Figure 1:**
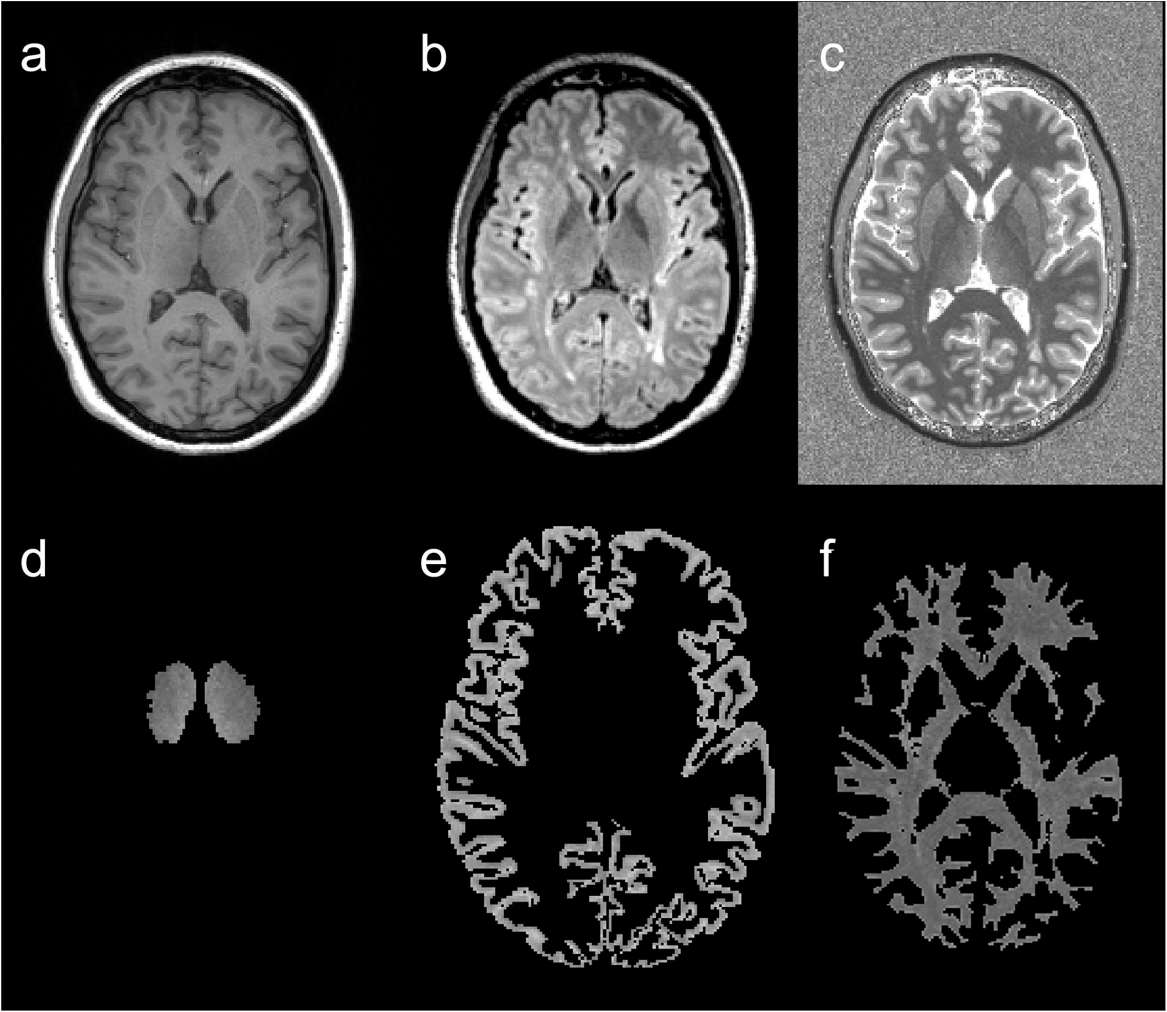
Example of image segmentation. T1w magnetization-prepared rapid gradient echo (MP2RAGE) **(a)**, fluid attenuated inversion recovery (FLAIR) **(b)**, and MP2RAGE **(c)** images and corresponding segmentation results of an MS patient's T1 relaxation times (T1-RT) map: thalamic T1-RT map **(d)**, cortical T1-RT map **(e)** and white matter T1-RT map **(f)** with removed lesions (NAWM).

After the transformation to the MP2RAGE images, each segmented tissue mask was visually inspected by an experienced neuroradiologist and manually corrected if necessary. Subsequently, the binary tissue masks (cortex, right and left thalamus, NAWM) were multiplied with the MP2RAGE image and additionally thresholded at T1-RT <3,500 ms to remove voxels that were mistakenly placed in the CSF (23). Finally, mean T1-RT was calculated for each tissue type. An example of tissue segmentation is displayed in Figure 1.

FSL's SIENAX was used on T1w MPRAGE images after lesion filling to obtain normalized brain volumes (NBV), GM, and WM volumes (GMV and WMV) for each subject (24).

### Statistical Analysis

Statistical analysis was performed using SPSS IBM SPSS 21 (IBM Corp., Armonk, NY, USA). Fisher's exact test was performed to compare categorical data between the groups. The Mann-Whitney U test was performed to compare patients with MS and healthy controls for demographic and imaging measures. Descriptive statistics were performed to obtain mean values, standard deviations, and ranges for all categories. Partial correlations with age, sex, and disease duration as covariates were obtained to characterize bivariate associations among imaging measures and clinical scores. Since multiple testing was performed, the Benjamini-Hochberg procedure was performed to adjust the *p*-values accordingly. *P*-values of <0.05 were regarded as statistically significant.

## Results

No significant differences were found between patients and healthy controls in terms of sex (*p* = 0.77) and age (*p* = 0.09). In respect to the imaging measures, patients with MS had significantly smaller NBV (1,466.07 ± 104.46 ml) compared to healthy controls (1,550.96 ± 64.34 ml; *p* =0.004), as well as WM (665.94 ± 65.00 vs. 707.76 ± 41.34; *p* =0.018) and GM volumes (800.13 ± 64.43 vs. 843.2 ± 46.23; *p* =0.015). Also, T1-RT was significantly increased in the NAWM in patients with MS (891.71 ± 35.88 ms) compared to T1-RT in the WM of healthy controls (854.76 ± 15.46 ms; *p* < 0.001). We found no significant differences for T1-RT in the left or right thalamus or the cortex between patients with MS and healthy controls. An overview is given in Table 1.

### Relationship Between Conventional Imaging Measures and Clinical Disability

In our study cohort, we found correlations between T2L volume and EDSS (*r* = 0.47, adjusted *p* (adj. *p*) = 0.01) as well as with the 25FW (*r* = 0.44, adj. *p* = 0.02). Additionally, NBV and GM volumes inversely correlated with EDSS (*r* = −0.38 and −0.45, adj. *p* = 0.02 and <0.01). We found no correlations between conventional imaging markers and SDMT.

### Relationship Between T1-RT and Clinical Impairment

**Figure 2:**
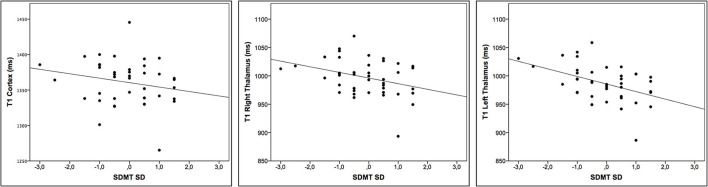
Correlation between T1 relaxation times and cognitive impairment. Scatter plots displaying the correlation between T1-RT in the cortex, right and left thalamus, and Symbol-Digit Modalities Test (SDMT) *SD* to age and education matched control cohort.

We found negative correlations between the T1-RT in the cortex, right and left thalamus and SDMT (*r* = −0.33, −0.37, and −0.50, adj. *p* =0.04, 0.03, and 0.01, respectively). The T1-RT in the NAWM did not correlate with any of the clinical tests. For a detailed overview, see Table 2 and Figure 2.

**Table 2:** Spearman rank correlation of MRI measures and clinical and cognitive test scores.

	**EDSS (*****n*** **=** **47)**		**SDMT (*****n*** **=** **44)**		**9HPT (*****n*** **=** **44)**		**25FW (*****n*** **=** **42)**	
	***r***	***p***	***r***	***p***	***r***	***p***	***r***	***p***
T2L volume	**0.47**	**0.01**	−0.32	0.05	0.20	0.15	**0.44**	**0.02**
NBV	**−0.38**	**0.02**	−0.18	0.18	−0.08	0.37	−0.15	0.30
GMV	**−0.45**	**<0.01**	−0.01	0.48	−0.39	0.06	−0.25	0.26
WMV	−0.10	0.42	−0.26	0.08	0.29	0.14	0.1	0.42
T1-RT Cortex	−0.09	0.38	**−0.33**	**0.037**	0.21	0.15	−0.02	0.46
T1-RT Right Thalmus	−0.30	0.05	**−0.37**	**0.03**	0.04	0.42	0.11	0.35
T1-RT Left Thalamus	−0.09	0.34	**−0.50**	**0.01**	0.23	0.17	0.20	0.24
T1-RT NAWM	0.06	0.36	−0.16	0.184	0.23	0.21	−0.23	0.23

*Significant correlations after adjusting to multiple comparisons using the Benjamini-Hochberg method are bold. EDSS, Expanded Disability Status Scale; SDMT, Symbol-Digit Modalities Test; 9HPT, Nine-Hole Peg Test; 25FW, Timed 25-Foot Walk; T2L, T2 lesion; NBV, Normalized brain volume; GMV, Gray matter volume; WMV, White matter volume; T1-RT, T1 relaxation time; NAWM, normal-appearing white matter*.

## Discussion

In this study, we used the MP2RAGE sequence for high-resolution T1-RT mapping to investigate T1-RT in the cortex, thalamus, and NAWM in patients with MS. T1-RT in NAWM were significantly higher than in the WM of healthy controls. We found no significant differences in T1-RT in the cortex or thalamus between patients with MS and HC. Additionally, we compared T1-RT and conventional MRI measures with clinical and cognitive disability. While T2L volumes correlated well with EDSS and 25FW, only T1-RT in the cortex and thalami correlated with the SDMT.

Previous studies suggested an important relationship between cortical and deep GM and cognitive impairment in patients with MS (25–27). Especially thalamic volume loss has been associated with impaired cognitive functioning (28, 29). In the present study, we found correlations between T1-RT in the cortex and the right and left thalamus and working memory and information processing speed, as measured by the SDMT. This extends the findings of recent publications which also found an association between T1-RT in GM structures and clinical disability. For example, Gracien et al. reported a strong correlation between T1-RT in the cortex and EDSS but only included 11 SP patients with MS and missed to investigate more detailed measures for cognitive and motor functioning (12). Furthermore, Gracien et al. did not find a correlation between thalamic T1-RT and EDSS.

In our study, we could not find an association between cortical or thalamic T1-RT and EDSS. Only T2L volumes, NBV and GM volumes correlated with EDSS (*r* = 0.42, −0.37, and 0.43, respectively, *p* < 0.05) which has been reported previously (30, 31). However, it has to be stated that the EDSS lacks specificity, especially when investigating cognitive impairment, and is well known for its intrinsic limitations. Besides its bias toward mobility and high inter-observer variability especially in the lower range, subtle clinical progression is often missed by its non-linear rating (32).

In a more detailed analysis, Steenwijk et al. investigated T1-RT in the cortex and thalami in 156 patients with MS and found a correlation between cortical T1-RT histogram skewness and impaired cognitive performance (14). However, they did not find correlations between mean T1-RT in the thalami and cortex and cognitive impairment. A possible explanation for this might be the different disease duration in our study cohort compared to the patients with MS included by Steenwijk et al. (6.5 ± 6.0 vs. 19.6 ± 6.9 years). As we know, T1-RT is affected by several microstructural changes in the WM and GM, such as the content of water, axonal density, gliosis, and iron accumulation (33, 34). With ongoing disease progression, a combination of these histopathological changes might counterbalance the T1-RT alterations leading to a reduced effect on T1 values. Of note, iron accumulation, which can be excessive in the GM of patients with MS, correlates negatively with T1-RT and can have a significant impact on mean T1-RT in the cortex and thalami in patients with longer disease duration (35). However, the literature is inconsistent regarding iron accumulation in the thalamus (31, 36, 37). As indicated by the results of Hagemeier et al., histopathological processes in the thalamus of patients with MS can be manifold leading to a challenging interpretation of imaging markers (37). For clarification, further emphasis should be laid on combining T1-RT with other non-conventional MRI techniques, such as magnetization transfer imaging (MTI), diffusion tensor imaging (DTI), and quantitative susceptibility mapping (QSM) to gain further knowledge on iron accumulation and myelin measurement *in vivo*. Undoubtedly, histopathological studies would provide the best evidence to verify these MRI techniques, but are naturally limited.

These counterbalancing effects might also be an explanation why we could not detect significant differences comparing T1-RT in the thalami and cortex between patients with MS and healthy controls. Only T1-RT in the NAWM was significantly higher compared to T1-RT in the WM of healthy controls. In comparison, some earlier studies reported increased T1 values in the cortex (12, 13, 38). While others did not find significant differences in T1-RT in the cortex and thalami between patients with MS and healthy controls (14, 26).

We applied quantitative T1 mapping to detect microstructural changes within the cortex and thalami. With the MP2RAGE sequence, it is possible to generate T1 maps (11 min and 17 s) with a high in- and through-plane resolution (1 mm × 1 mm × 1 mm) (16). Therefore, partial volume effects can be reduced to a minimum which can significantly corrupt the measures, especially due to cerebrospinal fluid in the subarachnoid space or the ventricles. Furthermore, T1-RT measured in MP2RAGE is highly reproducible both across and within the same subject which makes it also applicable for longitudinal studies. The scan time of 11 min and 17 s is comparable to other T1 mapping sequences applied in similar studies (13, 14).

There are some limitations to this study. Age and sex might influence T1 values of the thalami and cortex. To prevent a possible bias in our results, we included these two factors as covariates to remove their effect in our correlation analysis. Further, we are aware that there are different techniques to segment GM structures or T2L in Patients with MS. We used FreeSurfer and FSL's FAST to obtain tissue masks, which were additionally thresholded to exclude voxels that were mistakenly placed in the CSF. Further, the cortex segmentations were multiplied to obtain a consensus mask in the MP2RAGE space. Since each tissue mask was visually inspected by an experienced neuroradiologist and corrected if necessary, we are confident that our segmentations are most accurate. Healthy controls did not undergo cognitive testing. Therefore, we cannot determine whether an association between T1-RT in the cortex and thalamus and scores of the SDMT is a general finding or only specific to an MS study cohort.

In conclusion, our results suggested that T1-RT in the cortex and thalami correlate with the working memory and information processing speed, as measured by the SDMT, in Patients with MS. Therefore, T1-RT could help to gain further insight into histopathological changes within the brain associated with cognitive deficit. While cortical pathologies have been recognized to be associated with cognitive impairment, our results additionally highlight the influence of alterations in the thalami in patients with MS. For a better interpretation of our results, further emphasis should be laid on combining T1-RT with other non-conventional MRI techniques, such as MTI, DTI, and QSM. Whether this marker might also be useful in therapy monitoring and to capture disease progression would need to be assessed in longitudinal studies.

## Data Availability Statement

The raw data supporting the conclusions of this article will be made available by the authors, without undue reservation.

## Ethics Statement

The studies involving human participants were reviewed and approved by Ethics Committee Hamburg (Ethik-Kommission der Ärztekammer Hamburg). The patients/participants provided their written informed consent to participate in this study.

## Author Contributions

CT: conceptualization, methodology, software, validation, formal analysis, data curation, and writing—original draft and visualization. IH: methodology, validation, formal analysis, data curation, and writing—review and editing. J-PS and CH: resources, data curation, and writing—review and editing. MB: methodology, writing—review and editing, and supervision. JF: writing—review and editing, project administration, and funding acquisition. SG: methodology, software, investigation, data curation, writing—review and editing, supervision, and project administration. All authors contributed to the article and approved the submitted version.

## Conflict of Interest

The authors declare that the research was conducted in the absence of any commercial or financial relationships that could be construed as a potential conflict of interest.

## Publisher's Note

All claims expressed in this article are solely those of the authors and do not necessarily represent those of their affiliated organizations, or those of the publisher, the editors and the reviewers. Any product that may be evaluated in this article, or claim that may be made by its manufacturer, is not guaranteed or endorsed by the publisher.
